# Clinical outcomes of the chemo‐free approach in relapsed/refractory follicular lymphoma: A network meta‐analysis

**DOI:** 10.1111/bjh.70649

**Published:** 2026-07-01

**Authors:** Daniele Caracciolo, Maria Rita Lombardo, Gianluca Reitano, Cristina Napoli, Lucia Fiorillo, Chiara Callerame, Mario Garritano, Alessio Bulotta, Maha Munir, Giulia Pensabene, Emiliano Barbieri, Alberto Bavieri, Pierfrancesco Tassone, Pierosandro Tagliaferri, Stefano Luminari

**Affiliations:** ^1^ Department of Experimental and Clinical Medicine (DMSC) Magna Graecia University Catanzaro Italy; ^2^ Medical and Translational Medical Oncology Units, and Phase 1 Trial Center in Oncology and Onco‐Hematology AOU Renato Dulbecco Catanzaro Italy; ^3^ Hematology Division Azienda Unità Sanitaria Locale Istituto di Ricerca e Cura a Carattere Scientifico of Reggio Emilia Reggio Emilia Italy; ^4^ Doctorate School of Clinical and Experimental Medicine University of Modena and Reggio Emilia Modena Italy; ^5^ Department Chimomo University of Modena and Reggio Emilia Modena Italy

**Keywords:** anti‐CD20 antibodies, bispecific antibodies, chemotherapy‐free, follicular lymphoma, network meta‐analysis, relapsed/refractory

## Abstract

Relapsed/refractory follicular lymphoma (R/R FL) remains a clinically challenging condition, with progressively declining benefit across consecutive lines of therapy. While anti‐Cluster of Differentiation 20 (CD20) antibodies remain the therapeutic backbone, the optimal targeted or immune‐based partner remains undefined. A systematic review and network meta‐analysis (NMA) of 5 phase III and 1 phase II trials (2500 patients) evaluated seven anti‐CD20‐based combination strategies. Primary end‐points were overall survival (OS) and progression‐free survival (PFS); secondary end‐points included objective response rate (ORR) and grade ≥3 adverse events. Treatments were ranked using surface under the cumulative ranking (SUCRA) values. Immune‐activating strategies ranked highest across efficacy end‐points. Epcoritamab plus rituximab (R^2^) achieved the top SUCRA rankings for OS, PFS and ORR, while tafasitamab plus R^2^ consistently ranked second, with a better safety profile. R^2^ alone showed intermediate efficacy and good tolerability, whereas zanubrutinib plus anti‐CD20 demonstrated modest efficacy with lower toxicity. Bortezomib and copanlisib‐based combinations ranked lowest for efficacy, with phosphoinositide 3‐kinase (PI3K) inhibitor–based regimens showing the least favourable benefit–risk profile. Anti‐CD20 monotherapy was the safest but least effective option. This NMA indicates that immune‐centric, anti‐CD20‐anchored combinations represent the most effective chemotherapy‐free strategies for R/R FL. Combinations incorporating anti‐CD19 or CD20/CD3 bispecific antibodies appear to offer deeper disease control, particularly in high‐risk and rituximab‐refractory disease.

## Graphical abstract

Graphical overview of the network meta‐analysis (NMA) evaluating chemo‐free approaches for relapsed/refractory follicular lymphoma (R/R FL). Epcoritamab plus rituximab (R^2^) achieved the highest cumulative ranking probability (SUCRA) across efficacy end‐points but increases the risk of predictable toxicity over anti‐CD20 monotherapy. Created in BioRender.

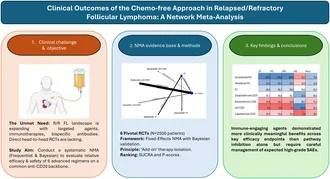

## INTRODUCTION

Follicular lymphoma (FL) is the most common low‐grade non‐Hodgkin lymphoma and is often characterized by an indolent course. Despite high initial response rates to first‐line chemoimmunotherapy, usually incorporating an anti‐CD20 monoclonal antibody, disease progression or relapse is expected in most cases as part of the disease trajectory.^1^, ^2^ For a relevant subset of patients, particularly those experiencing progression of disease within 24 months from diagnosis or beginning of first‐line therapy (POD24), outcomes are poorer, with shorter event‐free survival and reduced overall survival compared with patients with later relapse.^3^, ^4^ However, there is no single universally accepted standard of care beyond first relapse, and practice patterns vary widely across.^5^, ^6^

Anti‐CD20 monoclonal antibodies, such as rituximab and obinutuzumab, constitute the cornerstone of FL therapy across all disease phases, representing the backbone of most first‐line and subsequent treatment regimens; but the optimal agents to combine with this backbone remain insufficiently defined, particularly in the absence of evidence from direct comparison.^7^

Over the past decade, the therapeutic landscape has rapidly evolved with the development of several agents targeted at distinct biological pathways, designed to enhance or complement anti‐CD20‐mediated immune cytotoxicity: immunomodulatory drugs (IMiDs), phosphoinositide 3‐kinase inhibitors (PI3Ki), Bruton tyrosine kinase inhibitors (BTKi) and proteasome inhibitors (PI) demonstrated meaningful clinical activity in both earlier and later lines of therapy.^8^, ^9^, ^10^, ^11^

More recently, CD19‐directed therapies have further broadened the range of targeted partners available for combination with anti‐CD20 antibodies. Tafasitamab, an Fragment Crystallizable (Fc)‐enhanced anti‐CD19 monoclonal antibody, has been investigated in combination with lenalidomide and rituximab in patients with relapsed/refractory (R/R) FL and received regulatory approval in this setting, reflecting the growing clinical recognition of CD19 as a relevant therapeutic target in indolent B‐cell lymphomas.^12^ In parallel, bispecific antibodies targeting CD20 and CD3, such as epcoritamab, have demonstrated encouraging early efficacy in R/R FL, providing a novel T‐cell‐engaging immunotherapeutic approach.^13^

At the same time, novel immune‐based approaches such as Chimeric Antigen Receptor T cell (CAR‐T) based therapies are reshaping the treatment paradigm for FL experiencing quick disease progression, although phase III data for these treatment platforms remain limited and have not directly been compared with antibody‐based combination strategies.^14^, ^15^, ^16^

As a result, clinicians must make critical decisions on treatment sequencing and selection based only on cross‐trial comparisons, which are inherently hampered by differences in study design, eligibility criteria and end‐point selection.^17^, ^18^

In the absence of direct head‐to‐head randomized controlled trials (RCTs) comparing emerging chemo‐free regimens, clinicians face a significant evidence gap. Network meta‐analysis (NMA) addresses this by integrating direct and indirect evidence, allowing for a statistically sound comparison of regimens that have only been tested against common comparators (R^2^ or anti‐CD20 monotherapy).^19^ By modelling treatment effects across a connected network of trials, NMA can provide probabilistic rankings for each regimen in terms of key clinical outcomes, including overall survival (OS), progression‐free survival (PFS), response rates and safety, thus generating clinically meaningful insights to help therapeutic decision‐making when conventional pairwise meta‐analysis is insufficient^20^ and provide a treatment landscape analysis for the design of clinical investigations.

Therefore, to shed light on this complex scenario, we performed a comprehensive NMA of phase II–III randomized trials evaluating anti‐CD20 anchored combination strategies in R/R FL. By systematically comparing seven major classes of regimens, this analysis aims to clarify their relative efficacy and safety profiles, identifying immune‐ or targeted partners associated with the most favourable outcome.

## PATIENTS AND METHODS

### Systematic literature review

Based on the Preferred Reporting Items for Systematic Reviews and Meta‐Analyses (PRISMA) guidelines, bibliographic searches on PubMed, Embase, Cochrane Central Register of Controlled Trials and relevant abstracts and presentations of major meeting databases were carried out. Timeframe was set from January 1998 to December 2025. This systematic review was submitted to the international Prospective Register of Systematic Reviews (PROSPERO) platform and was registered with Prospero ID CRD420261280772.

### Data extraction and quality assessment

Data was extracted independently by two investigators (GR and MRL) using a predefined protocol/consensus. Hazard ratios (HRs) and 95% confidence interval (CI) were directly extracted for analysis. Both investigators assessed the risk of bias of the included studies by using the Cochrane risk of bias tool. Discrepancies were resolved through discussion with a third reviewer (DC) reaching consensus.

### Study selection

Inclusion criteria: (a) phase II or III RCTs; (b) enrolled patients with either histologically or cytologically confirmed relapsed/refractory FL; (c) compared two or more treatments; (d) reported detailed outcomes including PFS, OS, objective response rate (ORR) and treatment‐related adverse events (TRAEs).

The exclusion criteria were: (a) not randomized trials; (b) RCTs with no published results; (c) studies with unclear methodology; (d) trials including first‐line therapy; (e) studies focused on the analysis of selected population, on maintenance strategy or health‐related quality of life only.

### End‐points

Primary end‐points of the meta‐analysis were OS (time from randomization to death from any cause) and PFS (time from randomization to the date of objective disease progression or death from any cause). Secondary end‐points were ORR (sum of complete response and partial response) and serious adverse events (SAEs) (grade 3 [G3] or higher). The hierarchy of treatments was assessed using the surface under the cumulative ranking (SUCRA) curve. SUCRA values, ranging from 0% to 100%, represent the relative probability of a treatment being the best or among the best options. Within the frequentist framework, these values correspond to *P*‐scores; however, for clarity and consistency, they are referred to as SUCRA throughout. All rankings were interpreted strictly alongside their corresponding effect estimates (HRs for PFS and OS, odds ratios [ORs] for ORR, relative risks [RRs] for SAEs) and their 95% confidence (frequentist)/credible (Bayesian) intervals (CI and CrI, respectively).

### NMA software and analysis

NMA was performed using Stata software (StataCorp, version 17). Our analytical approach integrated both frequentist and Bayesian frameworks to ensure the robustness of the results.

The primary frequentist analysis was conducted using the network package (based on the *mvmeta* command). The network structure was visualized through the *network plot* command. Given the network's star‐shaped topology, characterized by the presence of single trials for most treatment comparisons and the absence of closed loops, there were zero degrees of freedom for both heterogeneity and inconsistency. Consequently, between‐study variance could not be statistically estimated, and formal testing for inconsistency (via node‐splitting or *ifplot*) was not applicable. Therefore, as mandated by the network structure, a fixed‐effects model was utilized. The network is built upon the principle of ‘add‐on’ therapy: within each included trial, the anti‐CD20 backbone is identical in both arms (e.g. anti‐CD20 + Agent X vs. anti‐CD20 monotherapy). This consistency justifies the synthesis of evidence across the network, as the relative effect of the ‘add‐on’ agent is isolated from the background effect of the CD20 immunotherapy. Treatments were ranked using the SUCRA curve, and a league table was generated using the *netleague* command to summarize all pairwise comparisons as HR, OR and RR with 95% CIs.

To validate the findings, a Bayesian NMA was performed using the ‘bayesmh’ command with Markov chain Monte Carlo (MCMC) simulations (30 000 iterations, 5000 burn‐in). Following the methodological considerations highlighted by Rosenberger et al.,^21^ we performed a sensitivity analysis comparing fixed‐effects and random‐effects models (using a weakly informative prior) to assess the impact of heterogeneity (*τ*) priors in our sparse network. Weakly informative normal priors [N(0, 10)] were used for treatment effects. Posterior probabilities of superiority were calculated to supplement the SUCRA rankings.

To mitigate the risk of over‐interpreting SUCRA rankings, we conducted sensitivity analyses by systematically excluding individual trials from the network (leave‐one‐out analysis). These analyses confirmed the stability of the treatment hierarchy, demonstrating that the clinical conclusions remain consistent despite the network's topology. To facilitate clinical interpretation, we utilized three complementary visual tools: the Network Plot (illustrating the landscape of available options), the Cumulative Probability Plots (SUCRA curves), and the netleague Table (presenting direct numerical comparisons between all pairs of treatments). Finally, a heat‐map graph was created to provide a visual synthesis of the overall effects across different outcomes.

## RESULTS

### Systematic literature review and studies included

**FIGURE 1:**
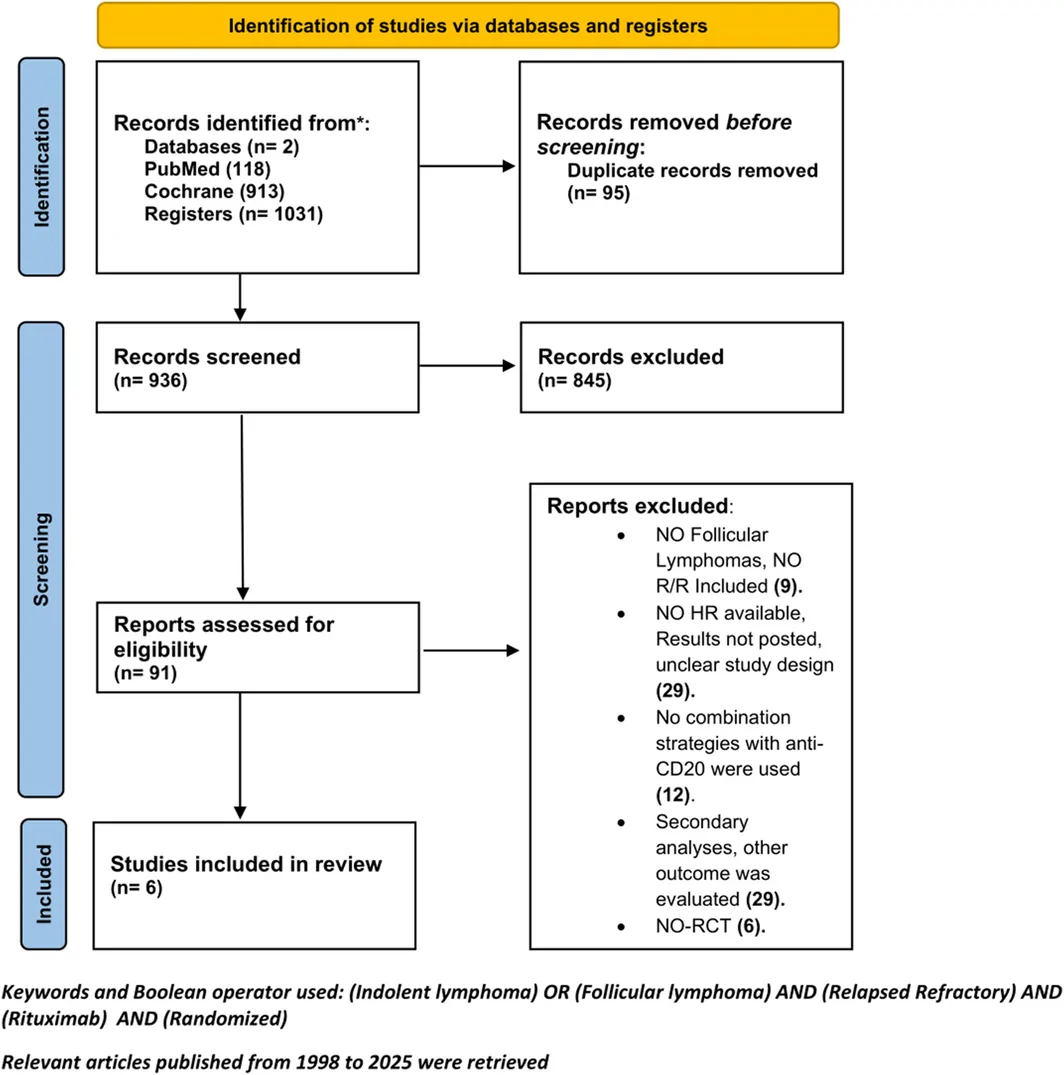
Preferred Reporting Items for Systematic Reviews and Meta‐Analyses (PRISMA) flow diagram used for studies identification and selection.

The PRISMA flowchart illustrating RCTs selection and the search strategy is shown in Figure 1. From 1998 to 2025, the systematic literature search initially identified a total of 1031 items including full articles and abstracts from meetings, which, after duplicates removal, were reduced to 936. Eight hundred and forty‐five studies were next excluded due to irrelevance after titles and abstract checking. Thus, 91 articles remained for eligibility assessment and full‐text evaluation. Of these, 85 were next excluded due to the following reasons: no‐FL, no relapsed/refractory (*n* = 9); secondary analysis, other end‐points evaluated (*n* = 29); antiCD20‐based combination not included as principal treatment (*n* = 12); no RCT (*n* = 6), no HR available, unclear study design (*n* = 29).

Finally, 6 trials including 2500 patients, met the inclusion criteria and were selected for NMA (Table 1).

**TABLE 1:** Baseline patient and clinical trial characteristics included in the network meta‐analysis.

Studies	AUGMENT		ROSEWOOD		LYM‐3001		CHRONOS‐3		inMIND		EPCORE FL‐1	
Treatment arms	Lenalidomide + rituximab (R^2^)	Rituximab	Zanubrutinib + obinutuzumab	Obinutuzumab	Bortezomib + rituximab	Rituximab	Copanlisib + rituximab	Rituximab	Tafasitamab + R^2^	R^2^	Epcoritamab + R^2^	R^2^
Patients (number)	147	148	145	72	336	340	184	91	273	275	243	246
Age, years, median (range)	64 (26–86)	62 (35–88)	63 (31–84)	65.5 (32–88)	57 (24–83)	57 (21–84)	63 (54–70)	62 (53–70)	64 (36–88)	64 (31–85)	60 (50–69)	63 (54–71)
Sex (male)	42%	54%	42%	54%	51%	40%	50%	56%	55%	54%	57%	56%
Baseline ECOG PS												
0–1	99%	99%	97%	99%	94%	94%	96%	99%	97%	97%	98%	97%
2	1%	1%	3%	1%	6%	6%	4%	1%	3%	3%	2%	3%
Bone marrow involvement	19%	17%	Not reported	Not reported	Not reported	Not reported	Not reported	Not reported	32%	33%	29%	28%
Ann Arbor stage												
Stage I–II	23%	31%	18%	17%	16%	18%	19%	16%	19%	18%	15% (only stage II)	18% (only stage II)
Stage III–IV	77%	69%	82%	83%	84%	82%	80%	81%	81%	82%	84%	82%
High tumour burden per GELF criteria	54%	48%	57%	56%	55%	53%	Not reported	Not reported	81%	84%	100%	100%
FL grade												
1–2	85%	83%	Not reported	Not reported	100%	100%	78.2%	73.7%	74%	74%	77%	67%
3a	15%	17%	Not reported	Not reported	0%	0%	21.8%	26.3%	25%	26%	21%	31%
Classic (5th edition WHO)	Not reported	Not reported	Not reported	Not reported	Not reported	Not reported	Not reported	Not reported	Not reported	Not reported	1%	1%
Missing	Not reported	Not reported	Not reported	Not reported	Not reported	Not reported	Not reported	Not reported	1%	Not reported	1%	1%
FLIPI												
0–1	29%	37%	Not reported	Not reported	23%	24%	Not reported	Not reported	21%	21%	26%	23%
2	31%	32%	Not reported	Not reported	36%	35%	Not reported	Not reported	29%	24%	33%	31%
3–5	39%	30%	53%	51%	41%	41%	Not reported	Not reported	50%	55%	41%	46%
Missing	1%	1%	Not reported	Not reported	0	0	Not reported	Not reported	0	<1%	<1%	0
B symptoms	9%	7%	Not reported	Not reported	Not reported	Not reported	Not reported	Not reported	23%	24%	Not reported	Not reported
Bulky disease (nodal or extra‐nodal ≥7)	25%	27%	16%	17%	Not reported	Not reported	Not reported	Not reported	Not reported	Not reported	19%	25%
N° previous therapies												
1	57%	54%	Not included	Not included	43%	40%	49%	47%	54%	55.6%	60%	57.5%
2–3	31%	34%	72%	75%	25%	26%	24%	26%	38%	36.7%	24%	25%
>3	11%	12%	28%	25%	32%	33%	26%	26%	8%	7.6%	16%	17.5%
Rituximab naive	15%	17%	0%	0%	57%	56%	1%	1%	Not reported	Not reported	0%	0%
Prior rituximab‐containing CHT regimen	73%	72%	100%	100%	43%	44%	99%	99%	66%	75%	98%	98%
Anti‐CD20 monotherapy	Not reported	Not reported	Not reported	Not reported	16%	18%	Not reported	Not reported	20%	19%	0%	0%
Anti‐CD20 monotherapy as only previous treatment	Not reported	Not reported	Not included	Not included	Not reported	Not reported	Not reported	Not reported	10%	7%	0%	0%
POD24	31%	34%	34%	42%	Not reported	Not reported	Not reported	Not reported	31%	32%	44%	38%
Double refractory (anti‐CD20 and alkylating)	Not reported	Not reported	Not reported	Not reported	Not reported	Not reported	Not reported	Not reported	Not reported	Not reported	37%	37%
Relapsed status to last therapy	Not reported	Not reported	Not reported	Not reported	Not reported	Not reported	Not reported	Not reported	54%	60%	Not reported	Not reported
Refractory status to last therapy	17%	14%	32%	40%	Not reported	Not reported	Not reported	Not reported	41%	35%	35%	33%
Refractory to previous anti‐CD20	Not reported	Not reported	54%	50%	Not reported	Not reported	Not reported	Not reported	43%	42%	43%	42%

*Note*: Comprehensive comparative profile of the randomized patient populations across the six pivotal clinical trials evaluating treatment regimens for relapsed/refractory follicular lymphoma. Data are stratified by individual treatment arms to summarize demographic parameters, baseline performance status, disease burden, staging and extensive prior systemic therapy exposure profiles. ‘Not reported’ indicates that the specific clinical variable was not explicitly documented or made publicly available by the original trial investigators within the published protocols or primary reports. ‘Not included’ specifies that the corresponding patient subpopulation was explicitly restricted by the mandatory inclusion/exclusion criteria of that respective trial's protocol.

Abbreviations: anti‐CD20, anti‐CD20 monoclonal antibody; CHT, chemotherapy; ECOG PS, Eastern Cooperative Oncology Group Performance Status; FL, follicular lymphoma; FLIPI, Follicular Lymphoma International Prognostic Index; GELF, Groupe d'Etude des Lymphomes de l'Adulte; POD24, progression of disease within 24 months from the onset of first‐line therapy; R^2^, lenalidomide plus rituximab; WHO, World Health Organization.

### Characteristic of included trials

This meta‐analysis included six RCTs (Table 1), five phase III and one phase II (Rosewood), all performed across multiple countries (Table 1). The mean age of patients ranged from 55 to 66 years, with a median age of 60 years and gender distribution was generally balanced. All studies enrolled patients with relapsed or refractory FL, across grades 1 to 3a, treated with various immunotherapy‐based regimens. Notably, the AUGMENT trial excluded patients who were refractory to prior rituximab therapy, whereas both the ROSEWOOD and EPCORE‐FL1 trials specifically enrolled patients with more advanced, high‐risk disease, including those refractory to rituximab. All trials included provided data on PFS, OS, ORR and safety outcomes, and were incorporated into the NMA.

We identified a total of 7 different treatment arms/regimens: lenalidomide + rituximab (R^2^), zanubrutinib + obinutuzumab, bortezomib + rituximab, copanlisib + rituximab, tafasitamab + lenalidomide + rituximab, epcoritamab + lenalidomide + rituximab, rituximab or obinutuzumab monotherapy. To simplify analysis, we decided to combine the arms containing Rituximab monotherapy and obinutuzumab monotherapy into a single ‘Anti‐CD20’ therapeutic arm.

### Quality assessment

**FIGURE 2:**
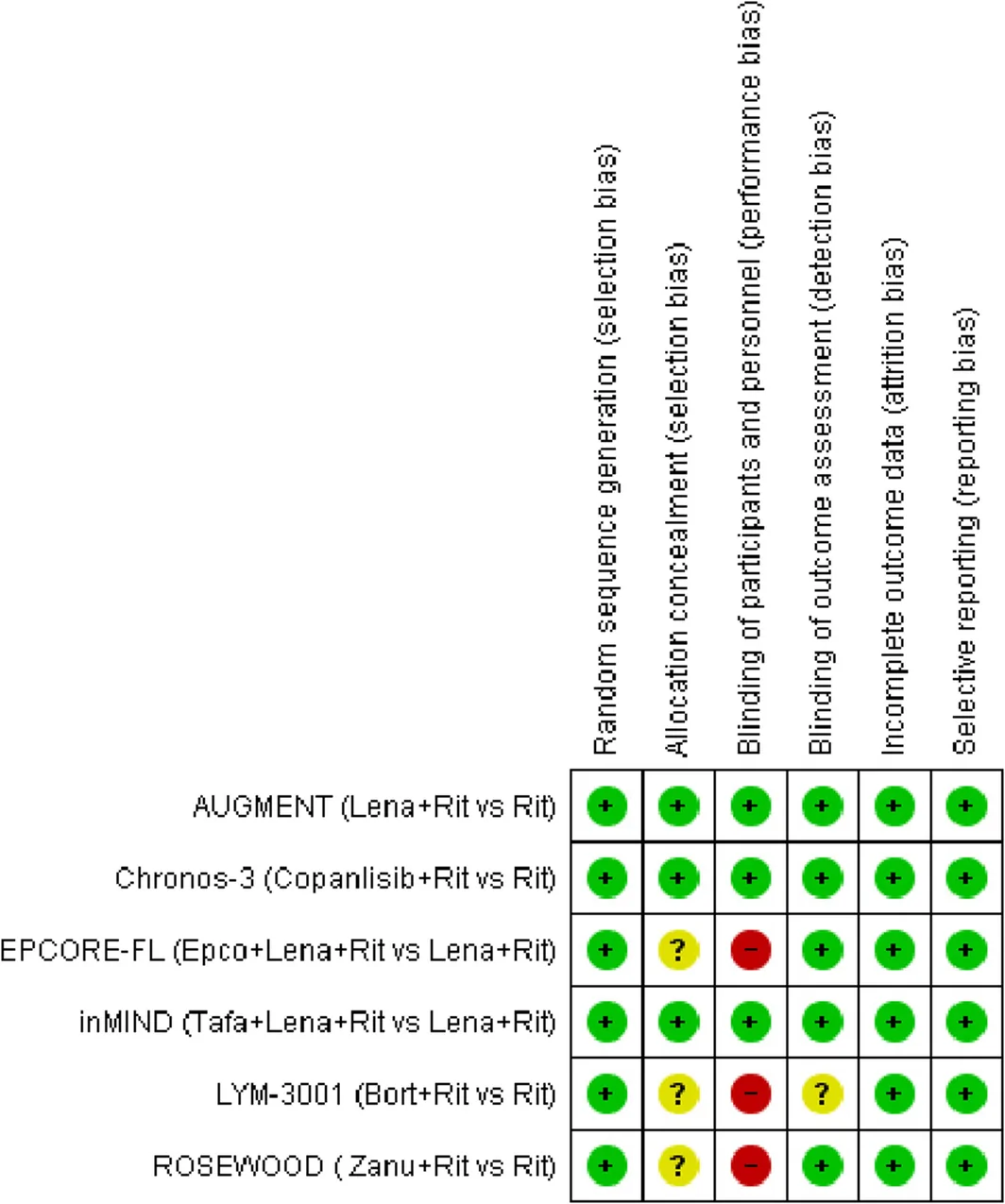
Quality assessment of included trials. Study‐level risk‐of‐bias assessment. Each row represents an individual trial, while columns correspond to specific bias domains.

The methodological quality of the included trials was assessed using the Cochrane risk‐of‐bias tool (Figure 2). The figure shows that most studies have a low risk of bias in key domains such as random sequence generation, allocation concealment and incomplete outcome data. However, some trials, notably EPCORE‐FL, LYM‐3001 and ROSEWOOD, exhibit unclear or high risk of bias related to blinding of participants and personnel (performance bias) and blinding of outcome assessment (detection bias), as indicated by the yellow and red colours in the figure.

### Network meta‐analysis

The primary NMA was performed using a frequentist fixed‐effects model. This approach was mandated by the network topology, which yielded zero degrees of freedom for both heterogeneity and inconsistency. Consequently, between‐study variance could not be statistically estimated, and the absence of closed loops precluded formal inconsistency testing. This structure justifies the fixed‐effects specification as the most appropriate and parsimonious method for evidence synthesis in this setting.

#### Overall survival

**FIGURE 3:**
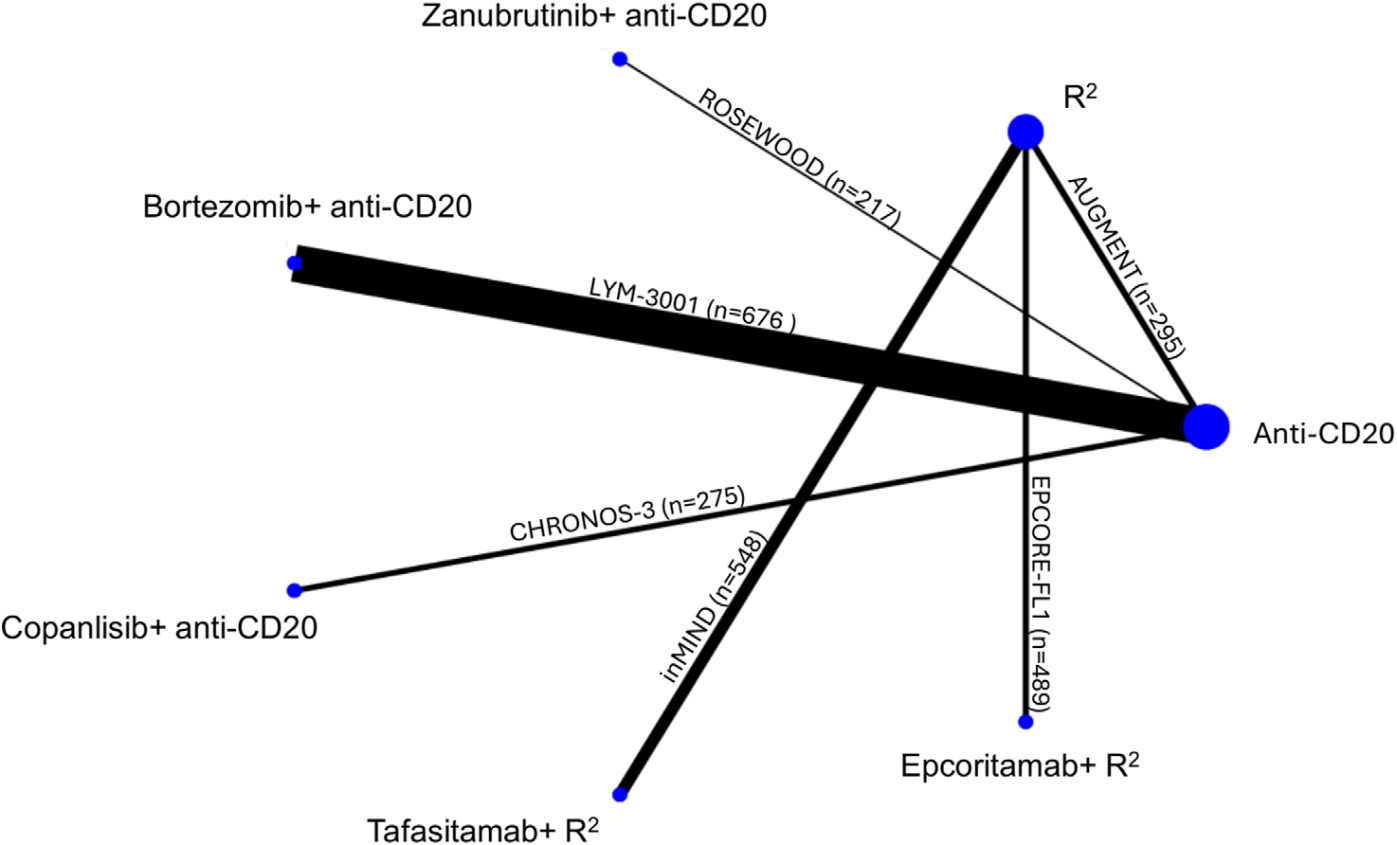
Network plot depicting the treatment regimens included in the network meta‐analysis (NMA) for overall survival (OS). Each node represents a therapeutic regimen, with the node size proportional to the total number of patients cross‐linked within the network for that specific treatment. Edges indicate direct head‐to‐head comparisons from randomized controlled trials; the line thickness reflects the cumulative sample size (number of patients) of the trials evaluating those treatment pairs. This visualization highlights the relative statistical weight and precision of each evidence link in the network.

The eligible comparisons of OS in the network plot including anti‐CD20, lenalidomide + rituximab (R^2^), zanubrutinib + anti‐CD20, bortezomib + anti‐CD20, copanlisib + anti‐CD20, tafasitamab + R^2^, epcoritamab + R^2^ are presented in Figure 3.

**FIGURE 4:**
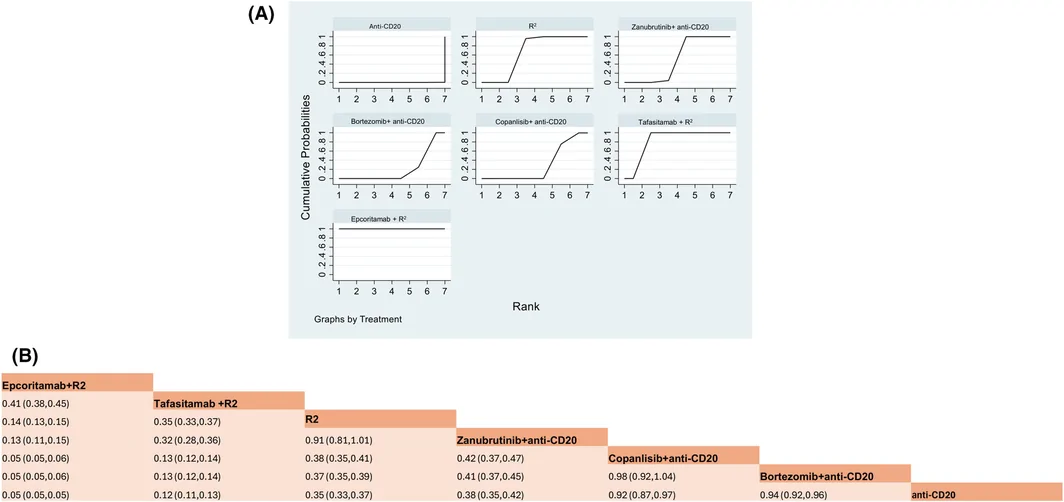
Network meta‐analysis for overall survival (OS). (A) Cumulative ranking plots based on the estimated surface under the cumulative ranking probabilities for OS. (B) Hazard ratios and 95% confidence interval for OS. The results are presented as column‐defined treatment versus row‐defined treatment. An hazard ratio (HR) <1.0 favours the column‐defined treatment.

The SUCRA probabilities (%) were ranked to compare effects of these agents on OS, and the results indicated that epcoritamab + R^2^ (100%) or tafasitamab + R^2^ (83.3%) were the most likely to improve OS (Figure 4A; Figure S1A). Specifically, epcoritamab + R^2^ demonstrated significant superiority over other regimens, as highlighted in the diagonal comparison of relative HRs in the netleague (Figure 4B). In particular, when compared with tafasitamab + R^2^ and R^2^, epcoritamab + R^2^ was associated with a statistically significant reduction in the risk of death of 59% (HR 0.41; 95% CI 0.38–0.45) and 86% (HR 0.14; 95% CI 0.13–0.15) respectively.

#### Progression‐free survival

The eligible comparisons of PFS in the network plot anti‐CD20, lenalidomide + rituximab (R^2^), zanubrutinib + anti‐CD20, bortezomib + anti‐CD20, copanlisib + anti‐CD20, tafasitamab + R^2^, epcoritamab + R^2^ are presented in Figure S2A.

**FIGURE 5:**
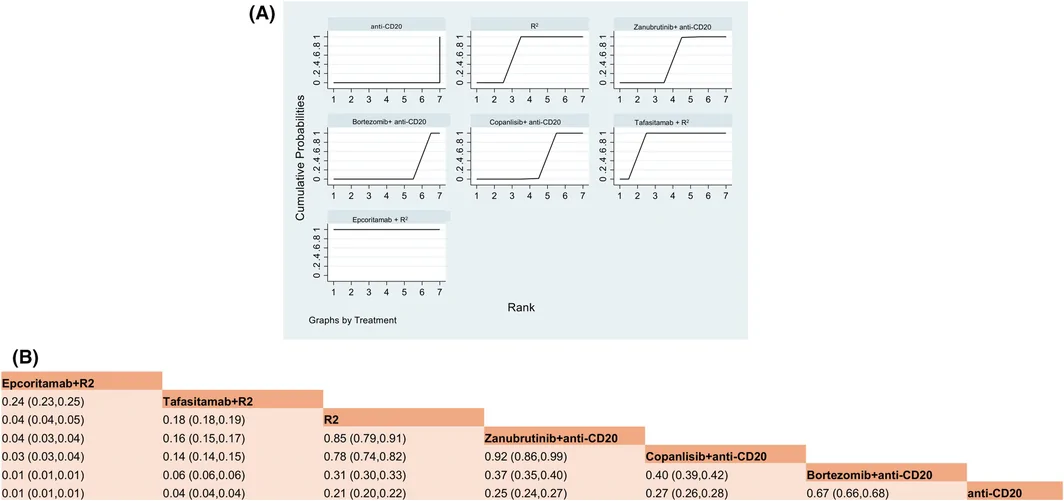
Network meta‐analysis for progression‐free survival (PFS). (A) Cumulative ranking plots based on the estimated surface under the cumulative ranking probabilities for PFS. (B) Hazard ratios and 95% confidence interval for PFS. The results are presented as column‐defined treatment versus row‐defined treatment. An hazard ratio (HR) <1.0 favours the column‐defined treatment.

The SUCRA probabilities (%) were ranked to compare effects of these agents on PFS, and the results indicated that epcoritamab + R^2^ (100%) or tafasitamab+R^2^ (83.3%) were the most likely to improve PFS (Figure 5A; Figure S2B). Specifically, epcoritamab + R^2^ demonstrated significant superiority over other regimens, as highlighted in the diagonal comparison of relative HRs in the netleague. Indeed, when compared with tafasitamab + R^2^ and R^2^ regimens, epcoritamab + R^2^ was associated with a statistically significant reduction of progression of 76% (HR 0.24; 95% CI, 0.23–0.25) and 96% (HR 0.04; 95% CI, 0.04–0.05), respectively (Figure 5B).

#### NMA for objective response rate

The eligible comparisons of ORR in the network plot are anti‐CD20, lenalidomide + rituximab (R^2^), zanubrutinib + anti‐CD20, bortezomib + anti‐CD20, copanlisib + anti‐CD20, tafasitamab + R^2^, epcoritamab + R^2^ presented in Figure S3A.

**FIGURE 6:**
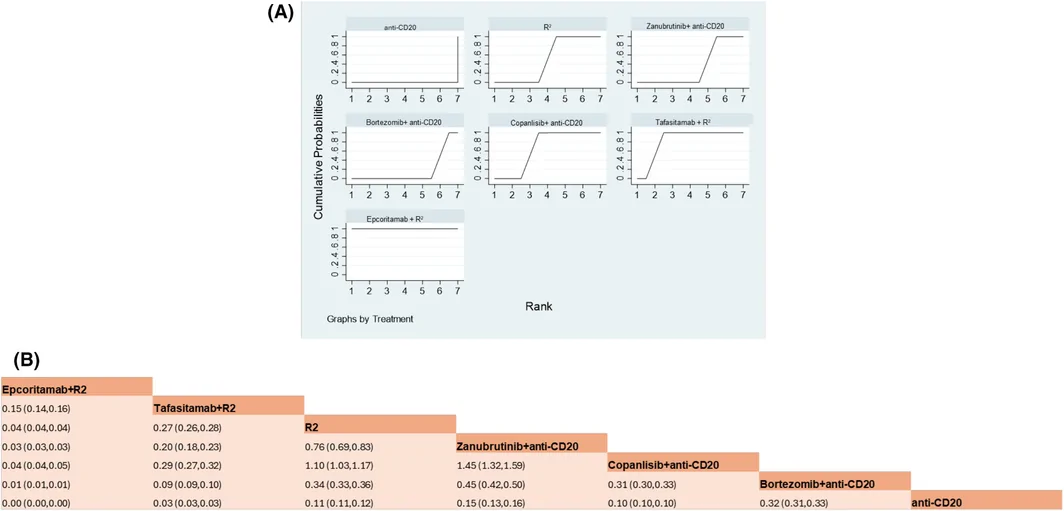
Network meta‐analysis for overall response rate (ORR). (A) Cumulative ranking plots based on the estimated surface under the cumulative ranking probabilities for ORR. (B) Odds ratios and 95% confidence interval for ORR. The results are presented as column‐defined treatment versus row‐defined treatment. Data express the odds of non‐response; therefore, an odds ratio (OR) <1.0 favours the column‐defined treatment.

The SUCRA probabilities (%) were ranked to compare effects of these agents on ORR, and the results indicated that epcoritamab + R^2^ (100%) or tafasitamab + R^2^ (83.3%) were the most likely to improve ORR (Figure 6A; Figure S3B). Specifically, epcoritamab + R^2^ demonstrated significant superiority over other regimens, as highlighted in the diagonal comparison of relative ORs in the netleague (Figure 6B). Epcoritamab + R^2^ demonstrated the best therapeutic effect on ORR, significantly reducing the likelihood of non‐response when compared to tafasitamab + R^2^ (OR 0.15; 95% CI, 0.14–0.16) and R^2^ (OR 0.04; 95% CI, 0.04–0.04) respectively.

#### Safety analysis

The eligible comparisons of SAE in the network plot anti‐CD20, lenalidomide + rituximab (R^2^), zanubrutinib + anti‐CD20, bortezomib + anti‐CD20, copanlisib + anti‐CD20, tafasitamab + R^2^, epcoritamab + R^2^ are presented in Figure S4A.

**FIGURE 7:**
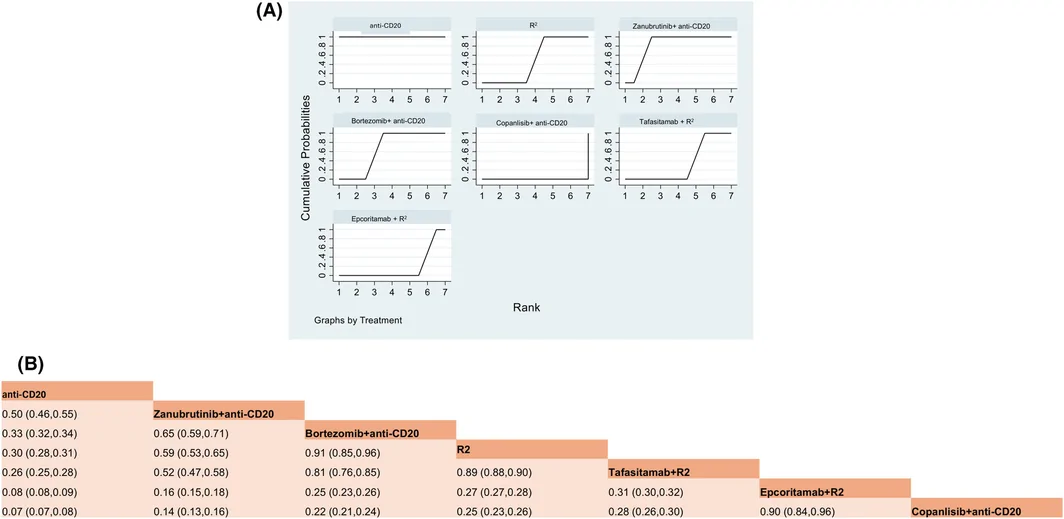
Network meta‐analysis for safety. (A) Cumulative ranking plots based on the estimated surface under the cumulative ranking probabilities for serious adverse event (SAE). (B) Relative risk (RR) and 95% confidence interval for SAE. The results are presented as column‐defined treatment versus row‐defined treatment. A RR <1.0 indicates a lower risk of serious adverse events, favouring the column‐defined treatment.

The results of the SUCRA probabilities (%) indicated that anti‐CD20 monotherapy was the most likely to improve tolerability (100%) followed by zanubrutinib combined with antiCD20 (83.4%); in contrast copanlisib + anti‐CD20 demonstrated to be the worse regimen in terms of safety (Figure 7A).

Specifically, in the pairwise comparisons anti‐CD20 demonstrated significant superiority over other regimens in terms of safety, as highlighted in the diagonal comparison of RRs (Figure 7B). Anti‐CD20 demonstrated the best safety profile when compared to zanubrutinib + anti‐CD20 (RR 0.50; 95% CI 0.46–0.55), bortezomib + anti‐CD20 (RR 0.33; 95% CI 0.32–0.34) and R^2^ (RR 0.30; 95% CI 0.29–0.31) respectively.

### Overall best regimen identification

**FIGURE 8:**
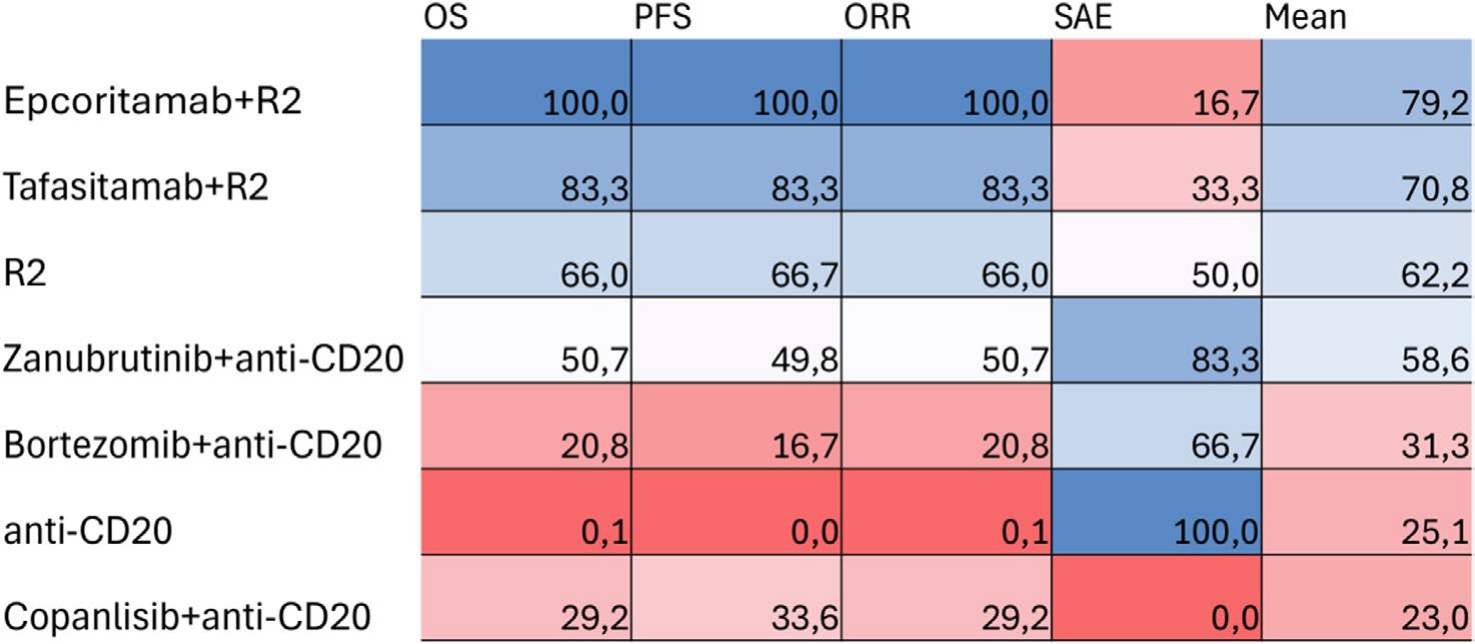
Heatmap of surface under the cumulative ranking (SUCRA) values for overall survival, progression‐free survival, overall response rate and serious adverse event analysed for each treatment regimen included in the analysis, ordered according to the mean SUCRA score (from the highest to lowest). Higher SUCRA values (approaching 100%), indicative of greater efficacy, are depicted in blue, whereas lower SUCRA values (approaching 0%) are shown in red, reflecting reduced efficacy.

Finally, among all regimens included in the NMA, we investigated which one scores as the overall best. To address this point, we assessed the SUCRA values for PFS, OS, ORR and SAE and estimated an average value to rank all the treatment options included in our analysis. According to average SUCRA values, the epcoritamab + R^2^ regimen achieved the highest score (average SUCRA: 79.2), followed by tafasitamab + R^2^ (average SUCRA: 70.8) and R^2^ (average SUCRA: 62.2) (Figure 8).

### Robustness and sensitivity analyses

To evaluate the robustness of our primary frequentist findings, particularly given the sparse nature of the treatment network, a Bayesian NMA^22^ was performed as a sensitivity analysis. Following the methodological considerations suggested by Rosenberger et al.^21^ regarding priors on heterogeneity, we compared the primary fixed‐effects results with a random‐effects model utilizing a weakly informative half‐normal prior for the heterogeneity parameter (*τ*) and a normal distribution [N(0, 10)] for treatment effects. Both Bayesian models confirmed the stability of the treatment ranking, particularly for the primary end‐point (PFS), where epcoritamab + R^2^ maintained a >90% probability of being the most effective regimen. However, the random‐effects model exhibited high autocorrelation and unstable convergence, likely due to the sparse nature of the treatment network and the negligible heterogeneity observed in the primary frequentist analysis. Consequently, the fixed‐effects Bayesian model was preferred for the final reporting, as it provided more parsimonious and stable posterior estimates (Table S1).

The EPCORE FL‐1 trial (epcoritamab + R^2^) has specific methodological and population characteristics that differ substantially from the other included studies, such as high percentage of patients with early relapse, POD24, or rituximab‐refractory disease which may finally influence the overall NMA results. Given these factors, EPCORE FL‐1 may disproportionately impact effect estimates and the relative ranking of treatments within the network, potentially introducing bias or heterogeneity that is not generalizable across the broader evidence base. To assess the robustness of the meta‐analytic findings and to evaluate the influence of this single study, a frequentist sensitivity analysis excluding EPCORE FL‐1 was conducted (leave‐one‐out analysis). This approach allows for verification of whether conclusions remain consistent when omitting a potentially influential and methodologically distinct trial, thereby strengthening the validity and interpretability of the overall results. Exclusion of the EPCORE FL‐1 trial led to a reshaping of treatment rankings across efficacy end‐points, underscoring the influential role of this study within the network: tafasitamab + R^2^ ascended to the first rank across all efficacy end‐points (OS, PFS and ORR: SUCRA 100%), R^2^ monotherapy saw an increase in its SUCRA values (e.g. OS SUCRA rising from 66.0% to 79.1%), while copanlisib + anti‐CD20 dropped significantly in the overall mean ranking (from 23.0% to 38.9% but shifting to the bottom of the efficacy table). Despite these shifts in individual positions, the overall clinical conclusions remain consistent in both direction and magnitude: combined immunotherapies (such as tafasitamab + R^2^ or R^2^) consistently outperform BTK or PI3K inhibitor‐based combinations and anti‐CD20 monotherapy in terms of survival and response. This stability confirms that the superiority of intensified immunotherapy regimens is a robust finding across the network and is not dependent on the inclusion of the EPCORE FL‐1 trial (Figure S5).

Finally, a third sensitivity analysis was conducted to verify that our methodological assumption of pooling rituximab and obinutuzumab under a single ‘anti‐CD20’ treatment arm did not bias the results. Importantly, removal of Rosewood trial (zanubrutinib + obinutuzumab) from the analysis, did not result in a relevant shift in treatment rankings (Figure S6).

These results validate the robustness of our findings and confirm that the observed efficacy hierarchy remains stable across different statistical frameworks and prior assumptions.

## DISCUSSION

The therapeutic landscape of R/R FL has evolved substantially over the past decade, with a progressive shift towards chemotherapy‐free strategies aimed at achieving durable disease control while preserving long‐term tolerability.^6^ Although chemoimmunotherapy remains highly effective in the front‐line setting, the relapsing nature of FL and cumulative toxicity associated with repeated cytotoxic exposure necessitate alternative approaches in later lines. Within this context, anti‐CD20‐anchored combinations with targeted or immune‐modulating agents have become central to treatment algorithms, though the optimal partner for anti‐CD20 antibodies remains incompletely defined.

This NMA provides a comprehensive comparison of all currently available chemo‐free combination regimens for patients with relapsed or refractory FL.

By leveraging direct and indirect comparisons, the analysis contextualizes relative efficacy and safety across therapeutic classes in the absence of head‐to‐head trials, with prespecified sensitivity analyses supporting the robustness of the findings.

Overall, regimens incorporating immune‐engaging mechanisms demonstrated more consistent and clinically meaningful benefits across key efficacy end‐points than pathway inhibition alone. Among all evaluated strategies, epcoritamab + R^2^ emerged as the most effective regimen, showing significant superiority for OS, PFS and ORR. Notably, the Bayesian sensitivity analysis fully confirmed these findings, demonstrating that the results remain robust regardless of the statistical framework employed.

It is critical to note that the numerical distance between SUCRA values does not necessarily correspond to a proportional difference in clinical efficacy. In our primary analysis, epcoritamab + R^2^ emerged as the top‐ranked regimen with a statistically significant reduction in the risk of death of 59% versus tafasitamab + R^2^ (HR 0.41; 95% credible intervals (CrI) 0.38–0.45) and 86% versus R^2^ (HR 0.14; 95% CrI 0.13–0.15). However, the distinction between subsequent top‐tier options is less pronounced. As shown by their similar trajectories in the cumulative ranking probability Plot, the exact ranking order and the differences between these regimens are not clear‐cut. This high degree of similarity indicates that while a mathematical ranking exists, these treatments may offer comparable clinical benefit. Consequently, the choice between such high‐ranking regimens should be guided not by rank alone, but also by safety profiles, costs and patient preference.

The phase III EPCORE FL‐1 trial provides key clinical context for these findings. In a population enriched for high‐risk features, including rituximab‐refractory disease and later‐line treatment, the addition of the CD20/CD3 bispecific antibody epcoritamab to R^2^ resulted in a marked improvement in ORR and a substantial reduction in the risk of progression or death compared with R^2^ alone. Although OS data remain immature, the magnitude and consistency of the PFS benefit underline the strong performance of epcoritamab‐based therapy observed in this analysis.

BTK inhibitor‐based combinations showed intermediate efficacy and acceptable tolerability but ranked consistently below immune‐engaging strategies, particularly in high‐risk disease. Furthermore, their apparent benefit may be overestimated in indirect comparisons due to the use of anti‐CD20 monotherapy as comparator in rituximab‐refractory populations, open‐label and phase II study designs, and the absence of an immunomodulatory backbone. In contrast, PI3K inhibitor–based regimens demonstrated limited efficacy and the least favourable safety profiles, in line with prior clinical experience and regulatory reassessments in indolent lymphomas.

Safety analyses revealed important class‐specific differences. Anti‐CD20 monotherapy showed the most favourable tolerability profile, followed by zanubrutinib + anti‐CD20, whereas copanlisib + anti‐CD20 ranked worst for safety. Immune‐engaging combinations were associated with higher rates of grade ≥3 adverse events but demonstrated overall manageable toxicity.

Sensitivity analyses confirmed the stability of the overall conclusions. Exclusion of EPCORE FL‐1 resulted in a reordering of treatment rankings, with tafasitamab + R^2^ emerging as the top‐ranked regimen for time‐to‐event outcomes, underscoring the influential role of EPCORE FL‐1 while confirming that immune‐centric strategies retain a comparative advantage. Pooling rituximab and obinutuzumab into a single anti‐CD20 category did not introduce meaningful methodological bias. Indeed, by removing ROSEWOOD trial no relevant shift in effect estimates or treatment rankings were observed, demonstrating that the observed clinical benefits are primarily driven by the experimental agents themselves or by the target population included in the study rather than the choice of the anti‐CD20 antibody backbone.

Indeed, a central limitation of our analysis lies in the heterogeneity of the clinical contexts in which the anti‐CD20 control arms, serving as the anchor for the network, were administered, including differences in prior rituximab exposure, refractoriness and baseline disease risk. For example, AUGMENT excluded rituximab‐refractory patients, whereas ROSEWOOD and EPCORE FL‐1 specifically enrolled rituximab‐refractory or double‐refractory populations (Table 1). Consequently, outcomes observed in anti‐CD20 monotherapy arms are not directly comparable and introduce unavoidable selection bias into indirect comparisons. Furthermore, several ongoing randomized trials will further inform and contextualize the findings of this NMA. Notably, the MAHOGANY trial is evaluating a head‐to‐head comparison of obinutuzumab plus zanubrutinib versus lenalidomide plus Rituximab in the second‐line setting, while the CELESTINO trial is comparing mosunetuzumab plus lenalidomide versus lenalidomide plus Rituximab. Such studies will further expand the body of direct comparative evidence and will serve as important reference points for interpreting the indirect comparisons from this NMA. Furthermore, while our analysis highlights the broad efficacy of certain regimens, a ‘one‐size‐fits‐all’ approach may not be ideal given the biological heterogeneity of FL. The trials included in this network, such as EPCORE FL‐1, often involve high‐risk populations characterized by early progression POD24; however, most current evidence lacks granular data regarding genetic aberrations (e.g. Enhancer of Zeste Homolog 2‐*EZH2, CREB binding protein‐ CREBBP* or Tumor Necrosis Factor Receptor Superfamily 14‐*TNFRSF14* mutations) or proliferation markers like Ki‐67.^23^, ^24^ Future research should aim for a stratified therapeutic approach where baseline molecular and cellular characteristics guide treatment selection. Integrating such biomarkers into large‐scale trials will be essential to transition from broad population‐based rankings to truly personalized treatment algorithms.

In addition, a limitation of our safety analysis is the exclusive focus on SAEs as the primary safety end‐point. While SAEs provide a robust measure of severe toxicity, they do not capture the full spectrum of lower‐grade, chronic adverse events that significantly impact patient quality of life. For instance, while regimens like copanlisib + anti‐CD20 showed high SAE probabilities, they are also associated with specific grade 1–2 toxicities, such as gastrointestinal issues, hyperglycaemia or hypertension, which may necessitate frequent dose interruptions or affect long‐term adherence. Since data on patient‐reported outcomes (PROs) and Health Related Quality of Life (HRQoL) were not uniformly available across the included trials, our findings should be interpreted as a measure of acute clinical severity rather than a comprehensive assessment of daily tolerability.

Finally, SUCRA‐based rankings should be interpreted with caution. While these values provide a useful hierarchy, they can be prone to overinterpretation because SUCRA reflects the position in a ranking distribution but does not convey the magnitude of the clinical benefit. A treatment may achieve a high SUCRA value due to the consistency of its data, even if its absolute clinical advantage over competitors is minimal. Consequently, our findings prioritize the integration of ranking probabilities with point estimates and the precision of the HRs to ensure clinical relevance is not overshadowed by numerical rank.

## CONCLUSIONS

Despite limitations of indirect comparisons, our study provides a structured comparative framework that helps contextualize efficacy signals across chemo‐free trials within their respective clinical settings.

Our findings support a risk‐adapted, immune‐centric approach to treatment sequencing in R/R FL. In patients with high‐risk features such as POD24 or rituximab‐refractory disease, intensification with epcoritamab + R^2^ appears justified by its substantial and consistent efficacy. For immune‐fit patients who are not candidates for bispecific antibody therapy or who experience disease progression after such treatment, anti‐CD19‐directed combinations incorporating immunomodulatory agents and anti‐CD20 antibodies retain a strong efficacy signal and long‐term disease control.

Conversely, in elderly or frail patients, lower‐intensity approaches such as BTK inhibitor‐based combinations may offer a more favourable balance between efficacy and tolerability. In later‐line settings or in selected patients with lower‐risk disease biology, immunomodulatory drug‐ or proteasome inhibitor‐based regimens may still play a role, primarily as disease‐controlling strategies rather than definitive long‐term control. Furthermore, there is a compelling need to evaluate the cost‐effectiveness of these regimens and to address the challenges of global access to immunotherapeutic drugs.^25^ This is particularly critical in countries lacking universal healthcare systems and in low‐to‐middle‐income countries, where resource constraints may limit the implementation of high‐cost therapies.^26^

Overall, this analysis reinforces the growing role of immune‐mediated strategies as the cornerstone of contemporary chemotherapy‐free treatment paradigms in R/R FL. However, future randomized studies with harmonized control arms, longer follow‐up and stratification by biological risk factors such as POD24 and rituximab refractoriness will be essential to refine treatment sequencing and to optimize the integration of emerging immune‐based therapies within clinical practice.

## AUTHOR CONTRIBUTIONS

**Cristina Napoli:** Visualization; validation. **Chiara Callerame:** Visualization. **Giulia Pensabene:** Visualization; data curation. **Maha Munir:** Visualization; software. **Daniele Caracciolo:** Conceptualization; methodology; writing – original draft; software; data curation; funding acquisition; formal analysis; writing – review and editing. **Emiliano Barbieri:** Visualization; investigation. **Alessio Bulotta:** Visualization. **Stefano Luminari:** Supervision; writing – original draft; visualization; writing – review and editing. **Pierosandro Tagliaferri:** Supervision; writing – original draft; writing – review and editing. **Maria Rita Lombardo:** Investigation; data curation; methodology; formal analysis. **Alberto Bavieri:** Investigation; visualization. **Lucia Fiorillo:** Visualization; validation. **Mario Garritano:** Visualization. **Gianluca Reitano:** Methodology; data curation; investigation. **Pierfrancesco Tassone:** Supervision; funding acquisition; resources.

## FUNDING INFORMATION

This manuscript has been supported by the Italian Ministry of Health PSC Salute 2014‐2020‐POS4 ‘Cal‐Hub‐Ria’ (grant T4‐AN‐09).

## CONFLICT OF INTEREST STATEMENT

The authors declare no competing financial interests.

## Supporting information


**Figure S1.** Table of results of SUCRA for OS.
**Figure S2**. (A) Network plot depicting the treatment regimens included in the NMA and (B) table of results of SUCRA for PFS.
**Figure S3**. (A) Network plot depicting the treatment regimens included in the NMA and (B) table of results of SUCRA for ORR.
**Figure S4**. (A) Network plot depicting the treatment regimens included in the NMA and (B) table of results of SUCRA for SAE.
**Figure S5**. Sensitivity analysis: Heatmap of surface under the cumulative ranking (SUCRA) values for OS, PFS, ORR and TRAE analysed for each treatment regimen, excluding epcoritamab + R^2^, ordered according to the mean SUCRA score (from the highest to lowest).
**Figure S6**. Sensitivity analysis: Heatmap of surface under the cumulative ranking (SUCRA) values for OS, PFS, ORR and TRAE analysed for each treatment regimen, excluding zanubrutinib + obinutuzumab, ordered according to the mean SUCRA score (from the highest to lowest).
**Table S1**. Bayesian sensitivity analysis results.

## Data Availability

All relevant data selected for the study are included in the article.
