# Supplementary material for: Clinical outcomes of the chemo‐free approach in relapsed/refractory follicular lymphoma: A network meta‐analysis
Source: Br J Haematol. 2026 Jul 1;209(3):874–86. doi: 10.1111/bjh.70649 (PMC13570175; doi:10.1111/bjh.70649)
Supplement: Supplementary file 1 [file BJH-209-874-s001.docx]

**Supplementary Figures**

**Supplementary Fig.1.** Table of results of SUCRA for OS

**Supplementary Fig.2**. (A) Network plot depicting the treatment regimens included in the NMA and (B) table of results of SUCRA for PFS

**Supplementary Fig.3.** (A) Network plot depicting the treatment regimens included in the NMA and (B) table of results of SUCRA for ORR

**Supplementary Fig.4.** (A) Network plot depicting the treatment regimens included in the NMA and (B) table of results of SUCRA for SAE

**Supplementary Figure 5. Sensitivity analysis:  Heatmap of surface under the cumulative ranking (SUCRA) values for OS, PFS, ORR and TRAE** analyzed for each treatment regimen, excluding Epcoritamab+R^2^ , ordered according to the mean SUCRA score (from the highest to lowest). Higher SUCRA values (approaching 100%), indicative of greater efficacy, are depicted in blue, whereas lower SUCRA values (approaching 0%) are shown in red, reflecting reduced efficacy.

**Supplementary Figure 6. Sensitivity analysis:  Heatmap of surface under the cumulative ranking (SUCRA) values for OS, PFS, ORR and TRAE** analyzed for each treatment regimen, excluding Zanubrutinib+Obinotuzumab, ordered according to the mean SUCRA score (from the highest to lowest). Higher SUCRA values (approaching 100%), indicative of greater efficacy, are depicted in blue, whereas lower SUCRA values (approaching 0%) are shown in red, reflecting reduced efficacy.

**Supplementary Table 1: Bayesian Sensitivity Analysis Results** This table reports the results of the Bayesian Network Meta-Analysis (NMA) performed as a sensitivity analysis to validate the primary frequentist findings. To account for potential network uncertainty while maintaining a conservative approach toward heterogeneity, a Bayesian framework with Rosenberger’s Half-Normal prior was applied.

Abbreviations**:** CrI, Credible Interval; HR, Hazard Ratio; SUCRA, Surface Under the Cumulative Ranking curve
